# High Parasitological Failure Rate of Visceral Leishmaniasis to Sodium Stibogluconate among HIV Co-infected Adults in Ethiopia

**DOI:** 10.1371/journal.pntd.0002875

**Published:** 2014-05-22

**Authors:** Ermias Diro, Lutgarde Lynen, Rezika Mohammed, Marleen Boelaert, Asrat Hailu, Johan van Griensven

**Affiliations:** 1 University of Gondar, Gondar, Ethiopia; 2 Institute of Tropical Medicine, Antwerp, Belgium; 3 Addis Ababa University School of Medicine, Addis Ababa, Ethiopia; Institut Pasteur de Tunis, Tunisia

## Abstract

**Background:**

Antimonials are still being used for visceral leishmaniasis (VL) treatment among HIV co-infected patients in East-Africa due to the shortage of alternative safer drugs like liposomal amphotericin B. Besides tolerability, emergence of resistance to antimonials is a major concern.

**Objectives:**

This study was aimed at assessing the clinical outcome of VL-HIV co-infected patients when treated with sodium stibogluconate (SSG).

**Methods:**

Retrospective patient record analysis of VL-HIV co-infected patients treated at a clinical trial site in north-west Ethiopia was done. Patients with parasitologically confirmed VL and HIV co-infection treated with SSG were included. The dose of SSG used was 20 mg Sb5 (pentavalent antimony)/kg and maximum of 850 mg Sb5 for 30 days. The clinical outcomes were defined based on the tissue aspiration results as cure or failure, and additionally the safety and mortality rates were computed.

**Results:**

The study included 57 patients treated with SSG and by the end of treatment only 43.9% of patients were cured. The parasitological treatment failure and the case fatality rate were 31.6% and 14.0% respectively. SSG was discontinued temporarily or permanently for 12 (21.1%) cases due to safety issues. High baseline parasite load (graded more than 4+) was significantly associated with treatment failure (odds ratio = 8.9, 95% confidence interval = .5-51.7).

**Conclusion:**

SSG is not only unsafe, but also has low effectiveness for VL-HIV patients. Safe and effective alternative medications are very urgently needed. Drug sensitivity surveillance should be introduced in the region.

## Author Summary

The co-infection of VL and HIV is a very challenging clinical problem especially in the East-Africa region. Though liposomal amphotericin B is the recommended treatment option for VL-HIV co-infection, it is often not available in practice in Ethiopia. Thus several patients are still being treated with antimonials that are infamous for their toxicity. In this study, we describe the results of such antimonial treatment in a series of 57 patients. The effectiveness of antimonials was found to be low and, in comparison to previous studies, declining. There is an urgent need to assure the availability of safer and more effective alternative medications for VL-HIV. It seems also wise to start a surveillance scheme for drug susceptibility in leishmania parasites as our results may relate to emerging antimonial resistance in the East-Africa region.

## Introduction

Visceral leishmaniasis (VL), also called kala-azar, is a vector-borne disseminated infection caused by the *Leishmania donovani* spp. complex, a protozoan parasite that predominantly affects tissue macrophages. Overt disease is lethal without treatment. The zoonotic form, with dogs as the main reservoir, is caused by *Leishmania infantum* and is found mainly in the Mediterranean basin and Latin America. The anthroponotic form is caused by *Leishmania donovani* and is prevalent in the Indian subcontinent (with estimated 300,000 cases/year) and East Africa (30,000 cases/year). Within East Africa, Sudan and Ethiopia are most affected [1]. Like most other neglected tropical diseases, VL has traditionally ranked low on the national and international health agenda [2]–[5].

HIV has been identified as one of the emerging challenges for VL control. HIV infection dramatically increases the risk of progression from asymptomatic *Leishmania* infection to VL and VL accelerates HIV disease progression [6], [7]. At the global level, the highest burden of VL-HIV co-infection is found in north-west Ethiopia, where up to 40% of VL patients can be co-infected with HIV [8].

Treatment of HIV co-infected individuals poses particular challenges with poor treatment response and recurrent relapse [9]–[12]. For over four decades, antimonials have been in use and still are the mainstay of therapy in East-Africa for immunocompetent individuals either alone or in combination with paromomycin [13]. Several studies demonstrated more than 90% effectiveness among VL patients without HIV [11], [14]–[17]. However, following reports of high rates of toxicity of antimonials in HIV-infected individuals [18], liposomal amphotericin B has been recommended as the preferential first line treatment for this patient group [13], [19]. Although the cost of liposomal amphotericin B has undergone substantial reductions for low income countries, there remain issues of availability of this drug in the public sector in several resource-constrained settings in East-Africa [20], [21]. In Ethiopia, the country with the highest co-infection rate globally, liposomal amphotericin B is in short supply. As a result, clinicians often still have to resort to antimonials for treating co-infected individuals, reserving the available liposomal amphotericin B for the most complicated or severe cases.

Besides poor tolerability, emergence of drug resistance is a major concern with antimonials, as has been witnessed in Bihar, India [22]. Whereas initial studies on antimonials reported relatively low rates of treatment failure in HIV-infected individuals in East-Africa [17], more recent retrospective cohort studies conducted in Ethiopia seemed to indicate relatively high failure rates [11], [14]. However, these reports came from settings where parasitological confirmation of diagnosis and treatment response was not systematically performed. Moreover, with the roll-out of Anti-Retroviral Therapy (ART) in East-Africa and better survival of HIV-infected patients, the VL patient population has gradually changed, with an increasing proportion of HIV patients presenting with VL relapse [23]. Data on efficacy and tolerability of antimonials in this patient population are very scarce.

Hereby, we report on the clinical outcomes in a cohort of adult VL-HIV co-infected patients recruited at the Leishmaniasis Research and Treatment Center (LRTC) of University of Gondar (UoG) Hospital, Ethiopia.

## Methods

### Ethics statement

Ethical approval was obtained for this study from the institutional review board of the University of Gondar. All data were analyzed anonymized.

### Study setting

The study was conducted at the LRTC in the UoG that is located in north-west Ethiopia close to the main VL endemic focus in the country. The LRTC was founded by the Drugs for Neglected Diseases initiative (DND*i*), and is now part of the governmental health system and is used as a clinical trial site. In addition to VL research, the center provides free VL treatment and care to all patients with leishmaniasis. Patients present to the center either spontaneously or are referred from other health institutes in the catchment area. Several international aid agencies including the World Health Organization (WHO), Médecins Sans Frontières (MSF) and DND*i* support VL treatment and care within Ethiopia, but shortage of anti-leishmanial drugs remains a frequent problem in clinical practice. Pentavalent antimonials and liposomal forms of amphotericin B, and more recently paromomycin, are the main drugs to treat VL. The combination regimen SSG and paromomycin was started as first line therapy for non-HIV VL cases at the site in September 2012. While liposomal amphotericin B is recommended for VL in HIV patients, due to the inadequate supply, it is often reserved for more severe cases such as patients with organ dysfunction. Miltefosine is infrequently available. As a result, most HIV co-infected VL patients are being treated with SSG.

### Study design and population

In November 2011 the LRTC initiated a clinical trial on the use of secondary prophylaxis to prevent relapse in HIV co-infected VL patients that is currently ongoing (http://clinicaltrials.gov/show/NCT01360762). All VL-HIV co-infected patients presenting at LRTC are screened for enrolment in this clinical trial and their findings and initial treatment responses are documented in individual patient record files. We reviewed these patient records and considered those who were initially started on treatment with antimonials between November 2011 and January 2013 as eligible for the present retrospective cohort study on safety and effectiveness of antimonials.

### VL diagnosis and treatment protocols

A VL diagnosis was suspected based on the WHO clinical case definition: prolonged fever, weight loss and splenomegaly in a patient from an endemic area or with a travel history. In patients who met this case definition we confirmed the VL diagnosis by microscopic detection of the parasite in tissue aspirates (spleen, bone marrow, lymph node). The parasite load was graded as 6+: >100 parasites per field; 5+: 10–100 parasites per field; 4+: 1–10 parasites per field; 3+: 1–10 parasites per 10 fields; 2+: 1–10 parasites per 100 fields; 1+: 1–10 parasites per 1000 fields and 0: 0 parasites per 1000 fields as viewed with a 100x oil-immersion lens [13].

Sodium stibogluconate (SSG) (at a dose of 20 mg Sb5 (pentavalent antimony)/kg for 30 days) was used for the initial treatment of these patients. The maximum SSG dose used was 850 mg Sb5/day, to avoid the toxicity related to high doses in HIV infected patients [24], [25].

### Treatment monitoring and follow-up

All patients were admitted to the LRTC and monitored for their treatment response using clinical and laboratory parameters. The main clinical parameters monitored during treatment were fever, appetite, fatigue, weight and spleen size. In addition, the patients were checked daily for new complaints especially related to adverse reactions. Blood chemistry and hematology tests were done weekly and a test of cure (ToC) by tissue aspiration and microscopic evaluation for parasites was done systematically at the end of SSG treatment (day 30). Electrocardiography was done based on the presence of cardiac symptoms. Treatment was extended for patients with a positive ToC at the end of SSG treatment. Those who i. showed a clinical response, ii. had a parasite load reduction of two log or more (“slow responders”) and iii. a parasite grade below 4 received a treatment extension with the same drug, while those who had less than two logs parasite reduction (“non-responders”) or a parasite grade of 4 or above were treated with an alternative drug (most commonly liposomal amphotericin B with or without miltefosine). While this was the principle followed, the shortage of medications often affected the practice. Depending on the parasite grade (high or low), ToC was repeated on day 15 or day 30 of the treatment extension with further extension of treatment when ToC was positive.

### HIV diagnosis and antiretroviral treatment

At LRTC provider-initiated testing and counselling for HIV is offered to all hospitalized patients. The HIV diagnosis is based on the national algorithm with two serial positive rapid test results; KHB (Shanghai Kehua Bio-engineering Co-Ltd, Shanghai, China) followed by STAT-PAK™ (Chembio HIV1/2, Medford, New York, USA). In case of discrepancy between the two tests, Uni-Gold™ (Trinity Biotech PLC, Bray, Ireland) is used as a tie breaker. As VL is considered a stage IV-defining illness in HIV patients [13], [19], all patients are given ART as soon as they were stabilized from their acute illnesses. ART regimens follow the national guidelines: tenofovir-lamivudine-efavirenz; zidovudine-lamivudine-efavirenz; or zidovudine-lamivudine-nevirapine [26]. Second-line ART consists of protease inhibitor-based combination regimens.

### Visceral leishmaniasis treatment outcomes

Initial cure is defined as a negative ToC at the end of the standard treatment [13]. A positive ToC indicates treatment failure, which consists of two types: slow-response or non-response. Clinical improvement (resolution of symptoms and signs such as fever, improvement in appetite, weight gain, gaining physical strength and regression of the spleen) with a reduction in parasite load by more than two logs without complete parasite clearance is defined as slow-response. If the parasite load remains the same or if the reduction is less than two logs, this is defined as non-response. SSG discontinuation as an outcome refers to permanent discontinuation of SSG due to intolerance.

Besides the initial treatment outcomes (at the end of the standard 30 days SSG treatment), treatment outcomes were also ascertained at the end of the entire treatment course (*end of treatment outcomes*), integrating eventual SSG extensions, or treatment changes for reasons of SSG intolerance or initial treatment failure.

Adverse reactions that required discontinuation (temporary or permanent) of the treatment were considered serious adverse drug reactions and included in the analysis. Reasons for interruption included clinical or biochemical pancreatitis; acute renal injury; or other severe conditions that were considered probably related to SSG like cardiotoxicity or worsening of bone marrow function. Clinical pancreatitis was defined as persistent vomiting, abdominal pain and raised serum amylase levels. Biochemical pancreatitis was defined as asymptomatic grade 4 serum amylase levels (using the Common Terminology Criteria for Adverse Events (CTCAE v 4.0) system [27]. Acute renal injury requiring SSG interruption was defined as acute increase of serum creatinine to more than 2 mg/dl (normal values 0.6 to 1.1 mg/dl). While some patients with toxicity improved after a temporary interruption (less than five days) and were able to continue with SSG, those with poor/slow recovery were shifted to other treatment regimens.

### Data collection and statistical analysis

We used a structured data collection format to extract information from the individual patient chart records. Socio-demographic data, weight, episode of leishmania, CD4 count, hematology and blood chemistry values and treatment outcome data were recorded. Count data were summarized as frequency (%), and numerical variables as median values with Inter-Quartile Range (IQR). To assess associations we computed Crude Odds Ratios (OR) with a 95% Confidence Interval (CI). For individuals with either initial cure or failure, the medians of within patient changes during treatment in laboratory and clinical parameters were compared using the Wilcoxon rank-sum test. *P*-values<0.05 were considered statistically significant. Statistical analysis was done using STATA 11 software.

## Results

### Demographic and baseline clinical characteristics

**Figure 1:**
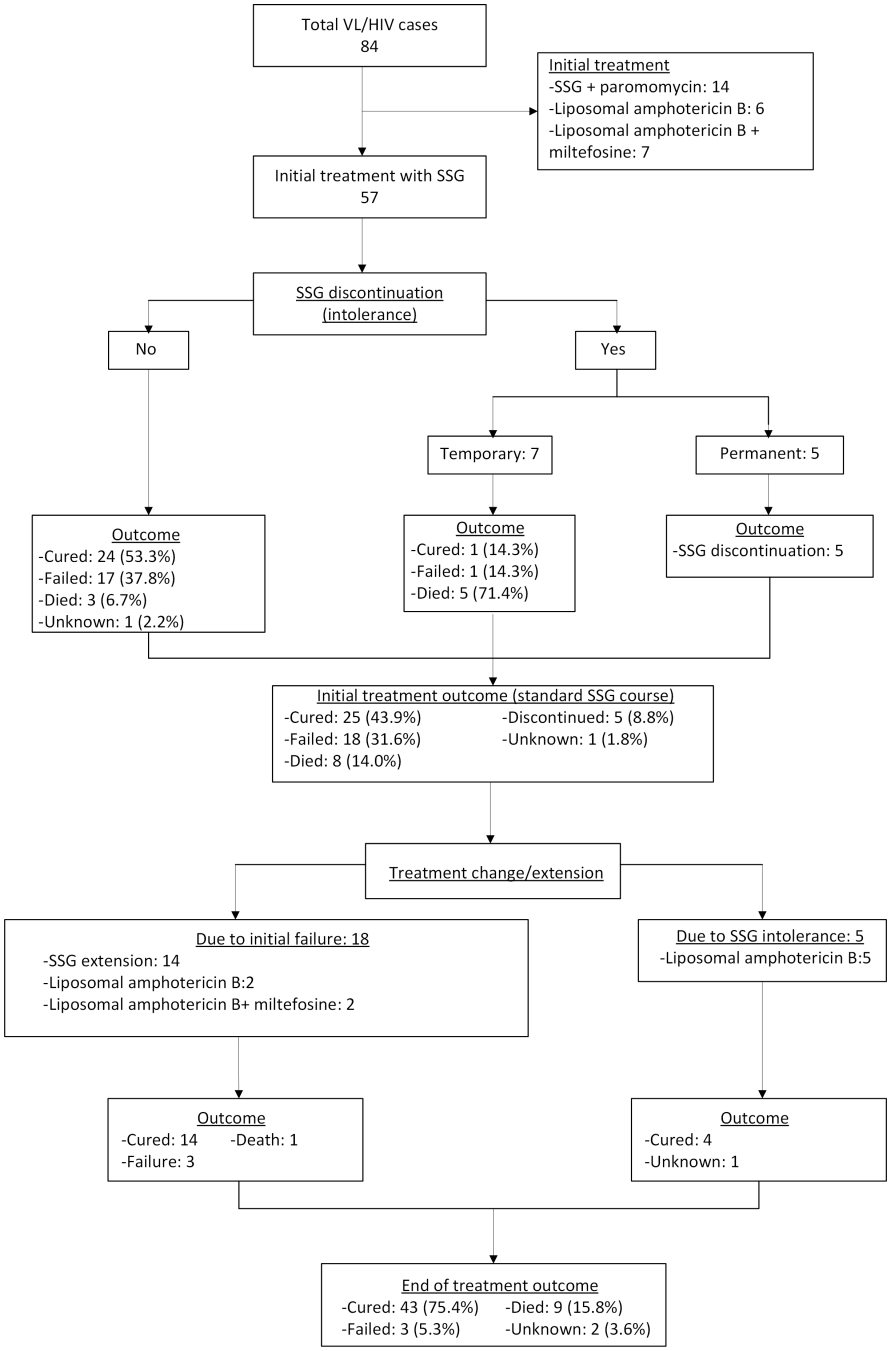
Flow diagram showing the number of patients in the study and their outcomes.

From a total of 84 HIV co-infected VL patients treated at LRTC during the study period, 57 received SSG as first line regimen. The others were treated with liposomal amphotericin B (6 patients), liposomal amphotericin B plus miltefosine (7 patients) or the SSG and paromomycin (14 patients) combination regimen, see Figure 1.

Of the 57 patients treated with SSG, 56 (98.2%) were male; the median age was 32 (IQR 28–36) years. While 33 (57.9%) were admitted for primary VL, 24 (42.1%) were relapses, and out of them 12 (50%) had more than two previous VL episodes. The majority were malnourished, with huge spleen and anemia (Table 1).

**Table 1:** Baseline characteristics of VL-HIV co-infected adults treated with SSG, Gondar, Ethiopia (2010-2012, N = 57).

Characteristic	Median (IQR) or N (%)
Sex	
Female	1 (1.8%)
Male	56 (98.2%)
Age (years)	32 (28–36)
CD4 cell count at VL diagnosis (cells/µL) (n = 53);	61 (35–101)
<200 cells/µL	48 (90.6%)
Baseline body mass index (kg/m^2^) (n = 53)	16 (15.6–17.4)
<18.5 kg/m^2^	45 (84.9%)
Palpable spleen	54 (94.7%)
Spleen size (if palpable) (cm)	10 (5–13)
Hematocrit (%) (n = 55)	28.4 (23.7–32.0)
<30%	35 (63.6%)
Tuberculosis treatment during current VL episode	3 (5.3%)
On ART at VL diagnosis	36 (63.2%)
Time on ART at VL diagnosis (weeks)	28.4 (14.1–43.6)
Not on ART at VL diagnosis	21 (36.8%)
Time to ART initiation (days) (n = 14)	19 (11–39)
Type of VL	
Primary	33 (57.9%)
Relapse	24 (42.1%)
>2 episodes	12 (21%)
Baseline parasite load from tissue aspiration (n = 56) ^a^	
>4	34 (59.6%)
≤4	22 (38.6%)
Site of tissue aspiration (n = 54)	
Spleen	42 (77.8%)
Bone marrow	10 (18.5%)
Lymph node	2 (3.7%)

ART: combination antiretroviral treatment; IQR: interquartile range; VL: visceral leishmaniasis; ^a^ in 2 it was not documented

Most patients (n = 36; 63.2%) were already on ART at the time of VL diagnosis. The median CD4 count was 61 (IQR 35–101) cells/µL, and 39 (73.6%) of them had a CD4 count less than 100 cells/µl. Most patients (n = 48; 90.6%) had a CD4 count less than 200 cells/µl. The leishmania parasite load in tissue aspirates was graded as +4 or above for 41/56 (73.2%) individuals (Table 1).

### Treatment and outcomes

Amongst the 57 patients starting antimonial treatment, SSG was permanently discontinued due to intolerance in five (8.8%) cases. In addition, seven (12.3%) patients temporarily interrupted SSG (for a maximum of five days) due to adverse reactions but re-instituted after stabilization. At the end of the standard SSG treatment course, 25 (43.9%) achieved initial cure while eight (14.0%) patients died during SSG therapy. One patient left the hospital against medical advice. There were 18 (31.6%) cases with initial treatment failure (positive ToC). Ten of these were slow responders while the eight others were non-responders.

For the 18 cases with parasitologically confirmed SSG treatment failure, treatment was extended beyond one month. A repeat course of SSG was used for 14 of these patients (for an additional 15 to 90 days). The other 4 were given liposomal amphotericin B (n = 2) or a liposomal amphotericin B and miltefosine combination (n = 2). For the five cases that permanently discontinued SSG due to intolerance, liposomal amphotericin B was given and four of them eventually got cured. At the global assessment made at the end of drug treatment, 43 of the 57 (75.4%) patients had achieved parasite clearance and were considered cured, three (5.3%) had treatment failure and nine (15.8%) died. The outcome was unknown for two (3.5%) individuals.

Serious adverse reactions were observed in 14 of the 57 patients (12 in the first month and 2 more during extension of treatment) mainly due to pancreatitis (n = 3), renal failure (n = 3) or both (n = 6). Cardiotoxicity and the combination of hepatitis and bone marrow suppression were observed in individual cases. Out of the nine deaths, five occurred after developing both pancreatitis and renal failure, and one after acute renal failure. The other three deaths were related to the presence of additional co-morbidities (malnutrition, sepsis and tuberculosis).

Table 2 shows the key clinical and laboratory parameters in relation with treatment response. While tissue parasite grading decreased more significantly in cured patients, there was a more pronounced increase in hematocrit and a greater reduction in spleen size in failure cases.

**Table 2:** Changes in clinical and laboratory parameters in relation to treatment response in VL patients treated with SSG, Gondar, Ethiopia (2010–2012).

	Cure			Treatment failure			P-value^b^
Variable	Baseline	End of treatment	Change^a^	Baseline	End of treatment	Change^a^	
Weight; kgmedian (IQR)	50 (43–52)(n = 27)	51 (43–53)(n = 27)	+1.5(−1;2)	49 (45–53)(n = 15)	52 (45–55)(n = 14)	+1(0;5)	0.53
Parasite loadmedian (IQR)	4 (2–5)(n = 29)	0(n = 29)	−4(2;5)	6 (5–6)(n = 18)	3 (1–4)(n = 17)	−3(2;4)	0.055
CD4 count; cells/µLmedian (IQR)	68 (36–151)(n = 28)	117 (58–305)(n = 26)	+59(−3; 174)	45 (35–64)(n = 17)	123 (99–250)(n = 14)	+62(23;215)	0.54
Spleen size; cmmedian (IQR)	8 (4.5–12)(n = 29)	4 (0–7)(n = 29)	−4(2;6)	10 (6–14)(n = 16)	2 (0–8)(n = 15)	−7(5;9)	0.014
Haematocrit; (%)median (IQR)	28 (25–32)(n = 28)	30 (25–37)(n = 28)	+3(−4; 7)	26 (22–33)(n = 17)	36 (31–38)(n = 16)	+8(2;15)	0.042
Absolute neutrophil count;count; median (IQR)	600 (430–1050)(n = 20)	1300 (800–1800)(n = 25)	+720(−100; 1200)	1000 (700–1800)(n = 11)	1650 (1300–2250)(n = 16)	+700 (0;1100)	0.98

IQR: interquartile range.

a Median of within patient change during treatment.

b Comparing median of within patient changes in individuals with cure or failure using the Wilcoxon rank-sum test.

### Risk factors associated with treatment failure and death

**Figure 2:**
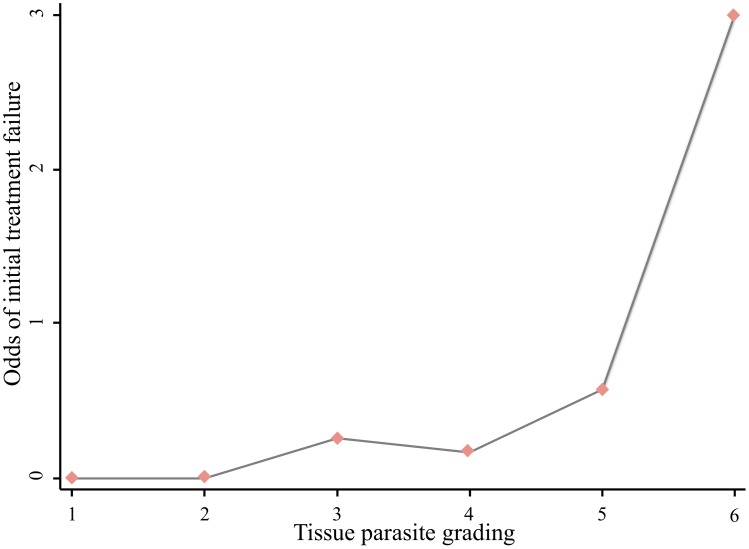
Association between the grade of *Leishmania* tissue parasite load at baseline and treatment failure in HIV co-infected adults treated with Sodium Stibugluconate (SSG), Gondar, Ethiopia (2010–2012, N = 56).

Although VL relapse cases and those on ART were more likely to fail, only high tissue parasite grading was significantly associated with treatment failure (OR = 8.9, CI = 1.5–51.7), Table 3 and Figure 2). Individuals with low CD4 counts (<100 cells/µl), malnourished patients (BMI <18.5 kg/m^2^), patients presenting with primary VL, patients having a large spleen size (≥10 cm) on admission and a higher tissue parasite load (≥4) were more likely to die, but these differences did not reach statistical difference (Table 3).

**Table 3:** Factors associated with treatment failure and death during treatment with SSG in HIV co-infected adults VL patients, Gondar, Ethiopia (2010-2012, N = 57).

	Treatment failure		Death	
	n/N (%)	Crude odds ratio (95% CI)	n/N (%)	Crude odds ratio (95% CI)
ART use at VL diagnosis				
No ART	4/21 (19.1)	1	3/21 (14.3)	1
On ART	14/36 (38.9)	2.7 (0.7–10.1)	5/36 (13.9)	0.97 (0.2–4.6)
VL episode				
Primary VL	8/33 (24.2)	1	6/33 (18.2)	1
Relapsed VL	10/24 (41.7)	2.2 (0.7–7.2)	2/24 (8.3)	0.40 (0.07–2.30)
Parasite load (N = 56)				
≤4	2/22 (9.1)	1	2/22 (9.1)	1
>4	16/34 (47.1)	8.9 (1.5–51.7)	5/34 (14.7)	1.7 (0.3–10.0)
Baseline CD4 count (N = 53)				
≥100 cells/µL	3/14 (21.4)	1	1/14 (7.1)	1
<100 cells/µL	14/39 (35.9)	2.0 (0.5–8.8)	6/39 (15.4)	2.4 (0.2–22.3)
Body mass index (N = 53)				
≥18.5 kg/m^2^	1/8 (12.5)	1	0/8 (0)	1
<18.5 kg/m^2^	15/45 (33.3)	3.5 (0.4–32.7)	8/45 (17.8)	-
Spleen size (N = 55)				
≤10 cm	6/26 (23.1)	1	2/26 (7.7)	1
>10 cm	10/29 (34.5)	1.8 (0.5–5.9)	6/29 (20.7)	3.1 (0.5–17.9)

ART: combination antiretroviral treatment; SSG: sodium stibogluconate; VL: visceral leishmaniasis; N: size of the subgroup; n: count in the subgroup.

## Discussion

This study showed high toxicity and very low effectiveness of SSG when used as a primary treatment option for VL-HIV co-infection. A 30-days drug course of SSG led to treatment failure in close to one out of three patients, an assessment based on parasitological data. SSG-related toxicity was common, probably contributing to death in six cases (i.e. 75% of the deaths). These findings indicate the urgent need of wider availability of alternative and safer drugs such as liposomal amphotericin B for this specific population.

Previous studies from the region have reported initial failure rates of SSG in the range of 2.3% to 14.1% [11], [14], [16]. Several factors might have contributed to the higher frequency observed in this study. In contrast to most other studies, proportionally more patients on ART and/or (multiple) VL relapse cases, identified as risk factors for treatment failure, were included [23]. High baseline tissue parasite grade – the strongest risk factor in our study – was also relatively common. Third, the parasitological response was not systematically assessed in the other studies, which could have led to under-diagnosis of failure. Moreover, a maximum dose of 850 mg SSG was used in this treatment center for this group of patients due to the experiences of exacerbated toxicity with higher doses in other regions with a similar patient population [25]. Whether parasite drug resistance, as observed in India [22], played a role is currently unclear. Although the change in parasite grading was lower in failing patients compared to cured ones, the difference was not statistically significant. Drug sensitivity testing of the isolated parasites is currently underway.

Safety of SSG has always been a concern. However, limited studies addressed the experience in the treatment setting concerning the adverse effects of SSG in the region. Vomiting has been reported as a common adverse event in most of the studies ranging from 8% to 38% [14], [16], [17], though the underlying cause of the vomiting is not clearly addressed. A quarter of the patients in this study were suffering from adverse reactions that required either temporary or permanent discontinuation of the SSG. Pancreatitis and renal failure were found to be the serious adverse reactions that were probably the main causes of death necessitating the need to close monitoring during treatment. This implies that SSG should only be used in a setting where monitoring of renal functions and pancreatitis is possible. As electrocardiographic monitoring was not systematically done, the cardiotoxicity might be under estimated.

The case fatality rate during treatment (14%) is in the previously observed range, (6.8%–33.3%) [11], [28]. The risk factors for mortality identified in our study are in line with the previous observations.

The strengths of this study were that it was conducted in a dedicated leishmania treatment and research center where tissue aspiration is routinely performed in HIV co-infection, and standardized treatment protocols and data collection tools are in place. Limitations include the relatively small sample size, and the missing information for some laboratory tests. Moreover, HIV-1 viral load testing and electrolytes measurements could not be conducted.

The small sample size in this study did not allow for a thorough study of risk factors for treatment failure, and did preclude any control for confounding in multivariable analysis. We observed that patients with high parasite load tended to respond slowly or not. Extension of treatment (using antimonials or liposomal amphotericin B) beyond 30 days helped to increase the cure rate from 44% to 75%. Patients who received prolonged duration of treatment seem to have tolerated SSG. These few patients were also with better clinical situations (higher hemoglobin and better spleen regression) which can be a source of bias. High SSG toxicity was observed with the daily maximal dose limited to 850 mg. It should be noted that higher dose and prolonged therapy could be at the expense of increasing toxicity. Higher daily dose of antimonials should be discouraged for HIV co-infected patients given the high toxicity observed at the current dose. Case-by-case decisions on dose and duration of therapy need to take into consideration the parasite load at the time of diagnosis and safety issues.

The small sample size in this study did not allow for a thorough study of risk factors for treatment failure, and did preclude any control for confounding in multivariable analysis. We observed that patients with high parasite load tended to respond slowly or not. Extension of treatment (using antimonials or liposomal amphotericin B) beyond 30 days helped to increase the cure rate from 44% to 75% with minimal additional toxicity. Given the small sample size, the safety of extending SSG in individuals tolerating the first month of SSG remains to be confirmed. On the other hand, high SSG toxicity was observed with the daily maximal dose limited to 850 mg. Consequently, a higher daily dose of antimonials should in general be discouraged for HIV co-infected patients. Case-by-case decisions on dose and duration of therapy need to take into consideration the parasite load at the time of diagnosis and safety issues.

Patients with VL relapse and those developing VL while on ART tended to fail SSG treatment. With further expansion and access to ART in this region, such difficult to treat patients might gradually become more prevalent. Additional studies focusing on treatment in this patient group should be conducted. Despite ART, these patients had profound immune deficiency as seen from their CD4 cell level. Additionally, most patients failed SSG treatment while on ART showing the need for additional treatment strategies. Early screening and treatment or primary prophylaxis are possible options yet to be explored. The reason behind the better response in hematocrit and spleen regression in the treatment failure group may be related to the longer treatment period. Patients with better hematocrit may survive longer than the severely anemic ones, and face repeated relapses.

This study re-confirms that SSG is not safe in patients with HIV co-infection and additionally shows that its effectiveness is low - and potentially declining - in the region [29]. However, despite the recommendations in the national and international guidelines, SSG remains in use due to shortage of alternative safe and effective medications. Even with the price reductions and donation programs of liposomal amphotericin B, there is still a limited and irregular supply in Ethiopia. We call on all stakeholders to urgently take measures to ensure a stable access to liposomal amphotericin B for all high risk groups – including HIV co-infected patients – in which treatment with antimonials leads to unacceptable high rates of toxicity and/or failure. A clinical trial evaluating high dose liposomal amphotericin B or a combination with miltefosine in HIV co-infection is about to be initiated in Ethiopia. If found effective, ensuring the availability of these drugs should be a priority.

This study provides evidence of high treatment failure (failure of parasite clearance) and toxicity in HIV co-infected VL patients treated with SSG. Assuring availability of safer and more efficacious treatment options for HIV co-infected VL patients in this region needs urgent attention. Furthermore, the need for drug resistance surveillance is called upon.
